# Stress and long-term memory retrieval: a systematic review

**DOI:** 10.1590/2237-6089-2019-0077

**Published:** 2020-10-08

**Authors:** Cadu Klier, Luciano Grüdtner Buratto

**Affiliations:** 1 Departamento de Processos Psicológicos Básicos Instituto de Psicologia Universidade de Brasília BrasíliaDF Brazil Departamento de Processos Psicológicos Básicos, Instituto de Psicologia, Universidade de Brasília, Brasília, DF, Brazil.

**Keywords:** Long-term memory, memory retrieval, stress, cortisol

## Abstract

**Introduction:**

The experience of stressful events can alter brain structures involved in memory encoding, storage and retrieval. Here we review experimental research assessing the impact of the stress-related hormone cortisol on long-term memory retrieval.

**Method:**

A comprehensive literature search was conducted on PubMed, Web of Science and PsycNet databases with the following terms: “stress,” “long-term memory,” and “retrieval.” Studies were included in the review if they tested samples of healthy human participants, with at least one control group, and with the onset of the stress intervention occurring after the encoding phase and shortly (up to one hour) before the final memory test.

**Results:**

Thirteen studies were included in the qualitative synthesis (N = 962) and were classified according to the time elapsed between stress induction and memory retrieval (stress-retrieval delay), the stress-inducing protocol (stressor), the time of day in which stress induction took place, sex, and age of participants. Most studies induced stress with the Trier Social Stress Test (TSST) between 15 and 25 minutes before the final memory (mostly recall) test and showed significant increases in cortisol levels and memory impairment.

**Discussion:**

The reviewed studies indicate that stress does impair retrieval, particularly when induced with the TSST, in the afternoon, up to 45 minutes before the onset of the final memory test, in healthy young men. These results may inform future research on the impact of stress-induced cortisol surges on memory retrieval.

## Introduction

Stressful events are common in everyone’s life, inducing physiological changes, such as increased heart rate and sweating.^1^ Stressful events can also impact memory and reasoning. Due to their relevance, stress and its main hormone, cortisol, are widely investigated.^2 - 7^ Here we review studies on the effects of stress on memory retrieval, highlighting inconsistencies found in the literature and presenting suggestions for future studies.

### Stress response and memory retrieval

Stressful events activate both the sympathetic nervous system (SNS), leading to the release of noradrenaline and adrenaline (NA) into the bloodstream by the adrenal medulla, and also the hypothalamic-pituitary-adrenal (HPA) axis, leading to the secretion of glucocorticoids (cortisol) into the blood by the adrenal cortex.^3^ Stress-induced cortisol release can have a direct impact on the hippocampus and the amygdala, brain structures involved in memory and emotional processes.^8^ Cortisol can cross the blood-brain barrier and bind to glucocorticoid receptors in the hippocampus, thus modulating hippocampal function, and consequently, modulating encoding and retrieval of long-term memories.^9^

A considerable amount of research has investigated the impact of stress-related cortisol release on memory processing. Empirical evidence, however, has been inconclusive, with studies showing that episodic memory can be enhanced,^10 , 11^ impaired^12 - 15^ or remain unaffected^16 - 18^ after a stressful event depending on the timing of the event. When the stressor occurs just before the retrieval of consolidated information, memory performance is impaired,^7^ as in the case of the student who knows the subject but cannot remember its contents during a high-stakes test.

### Present study

In this study, we reviewed the literature published over the last 12 years on the impact of stress on memory retrieval, focusing on some key aspects, such as the time elapsed between stress induction and memory retrieval (stress-retrieval delay) and the protocol used to induce stress in the laboratory (stressor).

Stress-retrieval delay is crucial for understanding how stress modulates memory.^15^ The fast stress response occurs seconds after the stressor and involves the release of adrenaline, which increases alertness and facilitates memory encoding.^6^ The slow stress response, conversely, occurs several minutes after the stressor and involves the release of cortisol, which impairs the retrieval of consolidated memories.^6^ In fact, a recent meta-analysis has concluded that acute stress shortly prior to retrieval can significantly impair memory retrieval.^7^ Because stressful events prior to memory retrieval are fairly common (e.g., go blank during a speech), we restricted our systematic review to studies in which stress induction occurred shortly before retrieval.

Stressor type is also important, as some tasks are more effective than others at stimulating cortisol release and emphasize different aspects of the stress response (physiological vs. social). In one common protocol, the Cold Pressor Test (CPT), participants submerge their dominant hand into cold water for a short period of time.^19^ The cold water triggers the HPA axis, inducing the release of cortisol.^9^ By contrast, in the Trier Social Stress Test (TSST), the participant takes part in a task that includes giving a public speech and doing mental arithmetic aloud.^20^ While stress induction in the CPT is driven mainly by physiology, in the TSST stress induction is influenced by social evaluation and unpredictability.^21^ Given these differences in the nature of the stressors, we ask whether their impact on memory retrieval may also be different.

In addition to timing and stressor, we also classified studies according to the time of day in which the retrieval session took place (morning vs. afternoon), sex, and age of participants. These factors were deemed important because they are known to affect cortisol response and because previous studies reported conflicting results. For example, cortisol levels are high in the morning and lower in the afternoon.^22^ However, stress-related memory impairment appears not to vary with time of day.^23^ Also, stress-related memory impairment seems to be sex- and gender-specific, having been observed mostly in young male participants^16^ : older participants are less affected, likely due to their lower responsiveness to circulating cortisol levels,^24^ and women are less affected depending on the phase of their menstrual cycle.^18 , 25^ We classified the search results along these factors (e.g., sex, age) in order to further explore their moderating influence on stress-related memory deficits.

## Method

The systematic review was conducted following the Preferred Reporting Items for Systematic Reviews and Meta-Analyses (PRISMA).^26 , 27^ PRISMA guidelines informed our search strategy, selection criteria, data extraction, and data analyses.

### Literature search

A comprehensive literature search was conducted by the first author in January 2020 on three databases: Web of Science, PsycNet, and PubMed. We looked for articles published from January 2008 to December 2019. On the Web of Science and PsycNet databases, the following keywords and Boolean terms were used: (“stress” AND “long-term memory” AND “retrieval”). The search on the Web of Science database was conducted on SCI-EXPANDED, SSCI, A&HCI, CPCI-S, CPCI-SSH, and ESCI indexes. On PubMed, the search was conducted with the following Medical Subject Headings (MeSH terms): (“Stress, Psychological” OR “Stress, Physiological” OR “Stress”) AND (“Memory, Long-Term” OR “Long-term memory”) AND (“Mental Recall” OR “Retrieval”).

### Inclusion and exclusion criteria

The following inclusion criteria were taken into consideration: experimental studies published in English, from 2008 to 2019, with samples comprised of healthy human participants, with at least one control group, and with the onset of the stressor intervention occurring after the encoding phase and shortly (up to one hour) before the final memory test. Literature reviews, experimental studies with animals or with human patients (e.g., chronic stress disorders), studies without stress manipulation or with stress manipulation occurring at a time other than before the final memory test (e.g., before or during encoding) were excluded.

### Data extraction and management

Eligible studies were selected based on title and abstract screening. Data from eligible studies were individually extracted and recorded in separate databases by the first author. Uncertainties were resolved through discussion with the second author. To improve objectivity, data from each study were organized through standardized forms containing the following categories: First author, year of publication, number of participants, participants’ age and sex, stressor, stress-retrieval delay, physiological measurement tools (e.g., cortisol saliva sampling), type of stimuli (e.g., word pairs, images, text), and type of final memory test (e.g., free recall).

## Results

**Figure 1:**
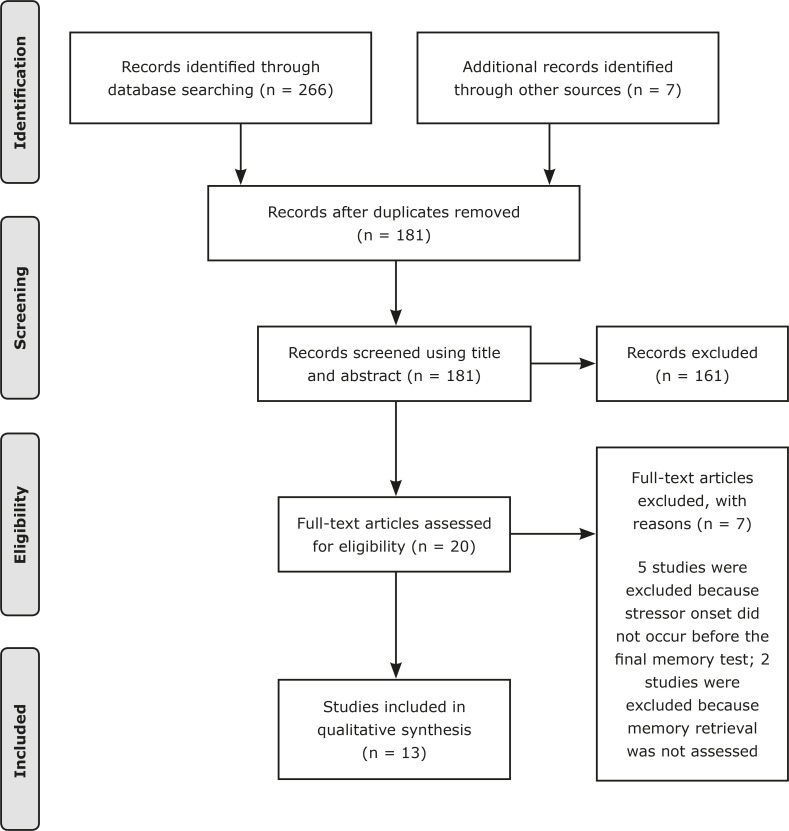
Systematic review flow diagram in accordance with the Preferred Reporting Items for Systematic Reviews and Meta-Analyses (PRISMA).

The initial search conducted on the three databases plus previously known articles yielded a total of 273 results: Web of Science (n = 125), PsycNet (n = 42), PubMed (n = 99), and other sources (n = 7). After removing duplicates, a total of 181 articles were screened (title and abstract) to check their relevance for this review. During the initial screening, 161 articles were excluded following the exclusion criteria. A total of 20 articles were assessed in full for eligibility. During full-text review, the first assessment was to check whether stress induction occurred before the final memory test. Five studies were excluded because stress onset occurred at a different moment of the experiment (e.g., during encoding or consolidation), and two studies were excluded because they tested cognitive functions other than long-term memory. Even though the literature on the impact of stress on memory performance is vast, only 13 studied survived our strict search criteria, with a total of 962 participants. Figure 1 shows the PRISMA flow diagram for study selection.

### Systematic review findings

Studies differed in several methodological dimensions, namely, types of stimuli (pictures, words, non-word syllable, text passage, search task and word-pairs), stressors (described below), stress-retrieval delay, time of day in which the retrieval session took place (morning vs. afternoon), sex, and age of participants (young vs. old participants). Table 1 summarizes the search results. A total of 25 experimental conditions (hereafter “experiments”) were described in the 13 reviewed studies. Eighteen out of the 25 experiments reviewed showed that stress affects memory retrieval. Data from two of these experiments showed memory enhancement, whereas data from the remaining 16 experiments showed memory impairment for participants in the stress group or for those classified as stress responders in a post-hoc analysis.

**Table 1:** Studies included in the systematic review

Study	Participant information			Stimuli used in encoding sessions	Retention interval	Stress protocol				Memory test type (time after stress onset)	Memory test results			
	
						Type of stressor (details)	Time of retrieval session	Change in cortisol levels (peak in min after onset)			CG × SG		RD × NR	
		
	M	F	Age range (mean)					M	F		M	F	M	F
Boehringer^12^	51	-	19-31 (24.6)	30 nouns	135 min	TSST	4-6 p.m.	- (25)	- (-)	Free recall (30 min)	SG impaired*	-	-	-
Goldfarb^13^ (1)	30	30	18-35 (23.0)	Multicued search task (“T” and “L”)	24 h	CPT (0-2 °C for 3 min)	Noon-6 p.m.	- (15)	- (15)	Search, stimulus-response (15 min)	Same	Same	-	-
Goldfarb^13^ (2)	30	30	18-35 (23.0)	Multicued search task (“T” and “L”)	24 h	CPT (0-2 °C for 3 min)	Noon-6 p.m.	- (15)	- (15)	Search, context memory (15 min)	SG impaired	SG impaired	-	-
Hidalgo^16^ (1)	27	25	56-76 (-)	30 color pictures	24 h	TSST	4-6 p.m.	Low (25)	No (-)	Free recall (25 min)	Same	Same	-	-
Hidalgo^16^ (2)	27	25	56-76 (-)	30 color pictures	24 h	TSST	4-6 p.m.	Yes (25)	No (-)	Recognition (25 min)	Same	Same	-	-
Hidalgo^16^ (3)	26	24	18-27 (-)	30 color pictures	24 h	TSST	4-6 p.m.	High (25)	High (25)	Free recall (25 min)	SG impaired	Same	-	-
Hidalgo^16^ (4)	26	24	18-27 (-)	30 color pictures	24 h	TSST	4-6 p.m.	Yes (25)	Yes (25)	Recognition (25 min)	Same	Same	-	-
Hupbach^10^	37	38	18-35 (-)	Neutral text with 30 ideas	24 h	CPT (0-2 °C for 2 min)	11 a.m.-4:30 p.m.	Yes (20)	No (-)	Free recall (immediate)	SG enhanced	Same	-	-
Larrosa^28^	6	12	18-40 (-)	5 pairs of nonsense syllables	3 days	CPT (0-4 °C for 1 min)	11 a.m.-5 p.m.	Yes (20)	Yes (20)	Cued recall (20 min)	SG impaired	SG impaired	-	-
Pulopulos^17^ (1)	38	38	56-76 (-)	30 color pictures	24 h	TSST	4-6 p.m.	High (25)	Low (25)	Free recall (25 min)	Same	Same	-	-
Pulopulos^17^ (2)	38	38	56-76 (-)	15 neutral words	24 h	TSST	4-6 p.m.	High (25)	Low (25)	Free recall (40 min)	Same	Same	-	-
Pulopulos^17^ (3)	38	38	56-76 (-)	2 stories (42 ideas)	24 h	TSST	4-6 p.m.	High (25)	Low (25)	Free recall (45 min)	Same	Same	-	-
Schonfeld^14^ (1)	36	36	- (23.2)	50 nouns and 50 pictures	24 h	TSST (core items)	1-6:30 p.m.	High (25)	Low (25)	Free recall (immediate)	Same	Same	RD/LD impaired^†^	RD/LD impaired^†^
Schonfeld^14^ (2)	36	36	- (23.2)	50 nouns and 50 pictures	24 h	TSST (core items)	1-6:30 p.m.	High (25)	Low (25)	Free recall (25 min)	Same	Same	RD/LD impaired^†^	RD/LD impaired^†^
Schoofs & Wolf^18^	-	36	- (24.7)	30 nouns	24 h	TSST	10-11 a.m.	- (-)	Low (10)	Free recall (25 min)	-	Same	-	Same
Schwabe^15^ (1)	20	20	- (23.6)	2 lists of 60 nouns	24 h	SECPT (0-2 °C for 3 min)	1-6 p.m.	No (-)	No (-)	Recognition (immediate)	Same	Same	-	-
Schwabe^15^ (2)	20	20	- (23.6)	2 lists of 60 nouns	24 h	SECPT (0-2 °C for 3 min)	1-6 p.m.	Yes (25)	Yes (25)	Recognition (25 min)	SG impaired^‡^	SG impaired^‡^	-	-
Schwabe^15^ (3)	20	20	- (23.6)	2 lists of 60 nouns	24 h	SECPT (0-2 °C for 3 min)	1-6 p.m.	No (-)	No (-)	Recognition (90 min)	SG impaired	SG impaired	-	-
Smeets^23^ (1)	17	21	18-25(19.9)	30 words	24 h	SECPT (0-2 °C for 3 min)	9-11 a.m.	Yes (20)	Yes (20)	Free recall (15-25 min)	SG impaired	SG impaired	-	-
Smeets^23^ (2)	17	21	18-25 (19.9)	30 words	24 h	SECPT (0-2 °C for 3 min)	2-6 p.m.	Yes (20)	Yes (20)	Free recall (15-25 min)	SG impaired	SG impaired	-	-
Smith^29^ (1)	48	72	- (20.1)	30 nouns and 30 images	24 h	TSST-G	3:30-5:30 p.m.	Yes (-)	Yes (-)	Free recall (immediate)	Same	Same	-	-
Smith^29^ (2)	48	72	- (20.1)	30 nouns and 30 images	24 h	TSST-G	3:30-5:30 p.m.	Yes (-)	Yes (-)	Free recall (20 min)	SG impaired	SG impaired	RP same as CG	RP same as CG
Szollosi^30^	28	35	19-27 (-)	40 word pairs	7 days	TSST	4-8 p.m.	Yes (-)	Yes (-)	Cued recall (30 min)	Same	Same	RD impaired	RD impaired
Zoladz^11^ (1)	38	55	- (19.45)	42 words	5 min	CPT (0-2 °C for 3 min)	Noon-6 p.m.	Low (22)	Low (22)	Free recall (immediate)	Same	Same	RD enhanced	Same
Zoladz^11^ (2)	38	55	- (19.45)	42 words	24 h	CPT (0-2 °C for 3 min)	Noon-6 p.m.	Low (22)	Low (22)	Recognition (22 min)	Same	Same	NR impaired	Same

Numbers in parentheses next to author name represent an experimental condition or group in the corresponding study.

- = not available; CG = control group; CPT = Cold-Pressor Test; F = female; LD = participants with low diastolic blood pressure; M = male; NR = cortisol non-responders; RD = cortisol responders; RP = retrieval practice condition; SECPT = Socially Evaluated Cold-Pressor Test; SG = stress group; TSST = Trier Social Stress Test; TSST-G = TSST group version.

* After controlling for changes in energetic and tense arousal, the association between increase in salivary cortisol and memory retrieval was no longer significant.

^†^ Only for negative items.

^‡^ Marginally significant.

### Stressors

Three stress induction protocols were identified in the search results, namely, the CPT, the Socially Evaluated Cold Pressor Test (SECPT) and the TSST. In the CPT, participants are told to submerge their dominant hand into cold water (0-3 °C) for a short period of time (1-3 min). The CPT triggers the HPA axis, leading to the release of cortisol.^9^ In the control task, participants submerge their dominant hand into warm water (around 30 °C) for the same amount of time. Four studies used CPT as the stress inducing protocol,^10 , 11 , 13 , 28^ with a total of six experiments and 246 participants (111 males, 135 females).

As in the CPT, participants in the SECPT also submerge their dominant hand into cold water. Unlike the CPT, however, participants in the SECPT are videotaped by the experimenter, who informs the participant that his/her reactions to cold water will be recorded for future analysis.^31^ Only two studies used SECPT as a stressor,^15 , 23^ with a total of five experiments and 196 participants (94 males, 102 females).

In the TSST, participants take part in a role playing task that simulates a job interview.^20^ They are told to prepare a 5-minute speech to be read in front of unfamiliar people. Following the speech, participants are also told to carry out mental subtractions aloud, being interrupted by the experimenters in case of mistakes. In the control task, participants perform similar activities but rather than giving a public speech, they write a note about a friend’s job interview and rather than doing mental subtractions aloud, they write the subtractions in a piece of paper. A total of seven studies used TSST as a stressor,^12 , 14 , 16 - 18 , 29 , 30 , 32^ with a total of 14 experiments and 520 participants (254 males, 266 females).

Reported cortisol levels varied across studies, being lower for older compared to younger participants, and lower for women compared to men. The two studies that tested older participants^16 , 17^ induced stress via the TSST. Despite smaller cortisol responsivity, older participants may experience higher levels of subjective stress in psychosocial tasks, such as the TSST.^9^

Some studies reported a post-hoc analysis whereby participants were grouped as responders (showing an increase of at least 2.5 nmol/l in cortisol levels) and non-responders (see Table 1 , last column). The aim was to assess whether cortisol responsivity (regardless of initial assignment to control vs. stress groups) was associated with memory impairment or enhancement. Two out of the five studies that performed this analysis found an impairment in delayed recall for responders after stress induction with TSST^14 , 30^ ; one study reported memory enhancement after stress induction with the CPT^11^ ; and the remaining two studies reported no difference between control and stress conditions after stress induction with the TSST.^18 , 29^

### Stress-retrieval delay

The time elapsed between stressor onset and the final memory test affected memory performance. In five experiments, memory was tested immediately after the stressor, and in 20 experiments there was a delay ( Table 2 ). When the criterial test occurred immediately after the stressor, participants in the stress and control groups produced similar results, except for one study^10^ that reported a significant memory enhancement for men. Another study^11^ showed, in a post-hoc analysis, that cortisol non-responders also had their memories enhanced. When the criterial test occurred 10-45 minutes after the stressor, results varied with either no change or an impairment in memory performance ( Table 2 ).

**Table 2:** - Summary of study characteristics included in the systematic review

Variable	No. experiments
Memory test type	
Recognition	6
Free recall	15
Cued recall	2
Search task	2
Participants’ characteristics	
Young men	19
Young women	19
Old men	5
Old women	5
Time of day	
Morning	2
Lunch time	2
Early afternoon	1
Late afternoon	11
Whole afternoon	9
Retention interval	
135 min	1
24 h	21
3 days	1
7 days	1
5 months	1
Stimulus type	
Pictures	8
Words	13
Non-word syllable	1
Text passage	2
Search task	2
Word pairs	1
Stress-induction protocols (stressors)	
TSST	14
CPT	6
SECPT	5
Stressor onset (min before criterial test)	
Immediate	5
15 to 25 min	15
30 to 45 min	4
90 min	1

TSST = Trier Social Stress Test; CPT = Cold-Pressor Test; SECPT = Socially Evaluated Cold-Pressor Test.

### Age

Only two studies addressed age as a factor, including older participants (56-76 years of age^16 , 17^ ). Both studies reported similar memory performance for both men and women under both conditions (stress or control). The level of cortisol increase in older participants was smaller than in younger participants, which could account for the lack of stress-related memory impairment in the old age groups.

### Sex

Only three studies reported significant differences in performance in the final memory test between men and women.^10 , 11 , 16^ When both sexes were in groups with successfully increased levels of cortisol, young men’s scores on the final memory test were lower than young women’s. Women in the luteal phase of the menstrual cycle are less responsive to surges in cortisol levels and their memory is less impaired than men’s, despite reliable cortisol increase following stress induction via TSST (the results reported by Schoofs & Wolf^18^ contrast with earlier results from the same laboratory^33^ ).

### Time of day

Cortisol levels are usually higher in the morning when compared to the afternoon. Most studies held the retrieval session in the afternoon, with only two studies having taken place in the morning.^18 , 23^ Smeets^23^ specifically assessed the role of time of day on stress-induced retrieval impairment by dividing participants into two groups, one with the session in the morning and the other in the afternoon. He found no difference in memory performance between the groups (morning or afternoon), suggesting that stress-induced retrieval deficits occur independently of time of day and that retrieval deficits are more related to the proportion of the increase in cortisol levels than to the absolute cortisol concentrations.

## Discussion

We reviewed studies assessing the impact of stress-induced cortisol increases on memory retrieval. In particular, we looked at differences in stressors, stress-retrieval delays, time of day of stress induction, sex and age of participants. The reviewed studies indicate that acute stress does impair retrieval, particularly when induced with the TSST in the afternoon up to 45 minutes before the onset of the final (recall) test in healthy young men.

The stress protocols used in the reviewed studies (CPT, SECPT, TSST) induced reliable increases in cortisol concentrations in both young men and women. These stressors, however, were less efficient with older participants of both sexes.^16^ The reason why older participants are less affected by these stressors is still an open question. One possibility relates to the fact that cortisol receptors in older participants are less densely distributed and less responsive in memory-relevant areas (hippocampus and prefrontal cortex) than in younger participants.^34 , 35^ This could partly account for the lower stress-induced memory impairments observed so far in older populations. Another possibility is that older participants are more habituated than younger participants to psychosocial stress. This possibility, however, is at odds with findings that older participants under stress (e.g., TSST) release more salivary alpha amylase, a SNS activity marker, than younger participants.^21^ In addition, older participants may experience higher levels of subjective stress than younger participants, possibly due to the unfamiliarity and unpredictability of the testing environment, which is more familiar to younger university students.^9^ Thus, it is not yet clear why stress-induced memory effects differ across age groups. Longitudinal studies (as opposed to the more common cross-sectional studies) coupled with novel stressor combinations (TSST plus SECPT, as proposed by Boyle et al.^36^ ) may induce more reliable cortisol increases in older participants and thus facilitate the assessment of cortisol-related effects in memory in this population.

Some stress-inducing protocols are more effective at eliciting HPA-axis responses than others. The TSST induces stronger cortisol and subjective stress responses than the CPT.^37^ In addition, the TSST induces more sustained stress responses than the SECPT.^38^ The subjective and SNS components of the stress response in the SECPT return to baseline by the time cortisol reaches peak levels. Adding the fact that the TSST has been studied for longer than the SECPT, it is understandable that most studies designed to assess the role of stress on memory chose to induce stress via the TSST. However, the SECPT has some attractive features, such as shorter application time (less than 3 minutes vs. 15 minutes in the TSST) and the presence of only one committee member (vs. three in the TSST). Similarly to the TSST, the SECPT has also been extended to group application and has been proven resistant to habituation effects.^39^ Thus, future studies using SECPT or hybrid TSST-SECPT protocols^36^ may help clarify the impact of HPA axis (and concurrent SNS) activation on memory retrieval.

Sex differences on memory performance were not evident in the reviewed studies. Women usually outperform men on episodic memory tasks.^40 , 41^ Despite superior memory, women are not immune to stress-related retrieval impairment,^29^ particularly if they are in the follicular phase of the menstrual cycle and not taking oral contraceptives.^7 , 21^ By contrast, women taking contraceptives or in the luteal phase of the menstrual cycle have their memories less affected by surges in cortisol levels.^7 , 18^ The lack of sex differences in stress-related memory impairments is consistent with the conclusions of a recent, focused review.^35^

Even though stress impairs retrieval,^7^ it does so more strongly for materials that have been encoded via repeated study than for materials that have been encoded via repeated retrieval.^29 , 42^ The latter strategy (retrieval practice) results in more enduring memories,^43^ even when compared to active encoding strategies.^44^ Because both memory and stress are ubiquitous in learning contexts,^45^ it is important to understand how they interact to improve or impair student outcomes. One direction for future research involves assessing whether the protection against the deleterious effects of stress afforded by retrieval practice is similar to different types of study materials (e.g., easy- vs. hard-to-learn materials), as difficult materials may be particularly affected by stress during high-stakes tests.

One limitation of the present review was its restricted scope. The focus on stress at retrieval was motivated by the ubiquity of stressors during recall tasks (e.g., during exams) and by the promising protective effects of retrieval practice on stress-related memory deficits. The restricted scope of our review, and consequent small number of eligible studies, also prevented us from conducting a quantitative analysis on the studies returned by the search. As the field progresses, future reviews may be able to quantify the individual roles of age, sex, and type of stressor on stress-related memory deficits.

In conclusion, we reviewed research assessing the impact of stress on memory retrieval. The reviewed studies indicate that stress impairs retrieval, particularly when induced with the TSST in healthy young men. These results may inform future research on the impact of stress-induced cortisol increases on memory retrieval.
